# Synthesis and Characterization of 2D-WS2 Incorporated Polyaniline Nanocomposites as Photo Catalyst for Methylene Blue Degradation

**DOI:** 10.3390/nano12122090

**Published:** 2022-06-17

**Authors:** Syed Shahabuddin, Shahid Mehmood, Irfan Ahmad, Nanthini Sridewi

**Affiliations:** 1 Department of Chemistry, School of Technology, Pandit Deendayal Energy University, Raisan, Gandhinagar 382426, Gujarat, India; 2 School of Bio-Chemical Engineering and Technology, Sirindhorn International Institute of Technology (SIIT), Thammasat University, PathumThani 12121, Thailand; shahid.mehmoodawan1@gmail.com; 3 Department of Clinical Laboratory Sciences, College of Applied Medical Sciences, King Khalid University, Abha 61421, Saudi Arabia; irfancsmmu@gmail.com; 4 Department of Maritime Science and Technology, Faculty of Defence Science and Technology, National Defence University of Malaysia, Kuala Lumpur 57000, Malaysia

**Keywords:** WS_2_, polyaniline, nanocomposite, photocatalyst, methylene blue

## Abstract

2D-WS_2_ incorporated polyaniline nanocomposites (WS_2_-PANI) with varying WS_2_ loadings were synthesized by a facile in situ oxidative polymerization technique which effectively promoted photocatalytic waste-water remediation using methylene blue (MB) as the probe molecules. The physicochemical properties of WS_2_-PANI (1–5) nanocomposites were investigated using multifarious techniques such as FT-IR, XRD, BET surface area, TGA, FESEM, and HRTEM. An electron microscopy analysis that was performed using HRTEM analysis confirm the layered structure of WS_2_ with periodic planes (100) separated by 0.27 nm. The photocatalytic performance of the WS_2_-PANI (1–5) for MB degradation performed under UV photo irradiation clearly showed that 2 wt.% WS_2_-PANI outperformed other variants with 93% degradation MB within 90 min. Furthermore, the catalytic material was reusable for five cycles without a significant loss of the catalytic performance.

## 1. Introduction

**Scheme 1:**
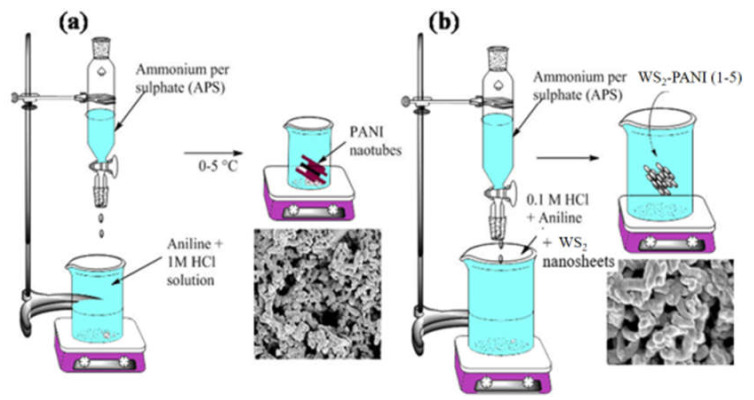
(**a**) Synthesis of PANI nanotubes; (**b**) WS_2_ incorporated PANI nanocomposite materials by oxidative polymerization technique.

Rampant industrialisation has accelerated the deterioration of aquatic ecosystems due to its discharge of highly noxious waste, and has thus received substantial global attention from researchers [1,2,3,4]. Major proportions of industrial wastage, including contaminates such as dyes, pesticides, and toxic heavy metals, are being regularly dumped into water bodies, thus making them the most vulnerable victim of environmental pollution [3]. Amongst the various contaminates, coloured organic dyes are the most notorious and non-biodegradable, [5] impacting aquatic flora and fauna via the reduction of solar radiation and thereby disrupting the photosynthetic reactions of the flora [6]. Additionally, these organic dyes from waste are hazards to human health [3,7]. In accordance with the estimation laid by the World Bank, a major chunk (10–15%) of organic colorants are frequently disposed via industrial waste effluents into multifarious aquatic environments, contributing to 17–20% of contaminates and hampering aquatic biosphere reserves [8,9,10]. Methylene blue (MB) (see Scheme 1) is a synthetic basic non-biodegradable dye and a key contributor to aquatic non-biodegradable pollutants. MB is frequently used in laser printing, textiles, and food; furthermore, these dyes are used as additives and are usually very stable, persistent, and immensely injurious to living beings in higher dosages leading to sever health consequences such as diarrhoea, tissue necrosis, jaundice, cyanosis, quadriplegia, and sometimes cancer [11,12,13,14,15]. Therefore, there is an urgent need for the development of effective techniques which can overcome the harmful consequences caused by these pollutants. Photocatalysis has emerged as one of the most effective techniques among the available methods for degrading toxic pollutants from waste water, and it operates by effectively detoxifying hazardous organic pollutants via solar or UV light [10,12,16].

Recently, nanomaterials clubbed with conducting polymers (CPs) have offered attractive alternatives to the pre-existing nanomaterials because of their adaptive surface chemistry, high mechanical tolerance, high surface-to-volume ratio, uniformly porous surface, and facile revivification under moderate conditions [17,18]. The CPs are composed of polymeric chains containing unsaturated bonds that form the π-conjugated systems responsible for well-defined optoelectronic and electrical properties. Recent CP nanocomposites such as polythiophene (PTh), polypyrrole (Ppy), polyaniline (PANI), polyethyleneimine (PEI), etc., have been in the spotlight due to their versatility and manifold applications in sensing, supercapacitors, battery materials, photocatalytic materials, photocatalysis, electrochemistry, and electroluminescence [10,17,19,20,21,22]. Amongst all these qualities, PANI are economical: they are synthesised by straightforward techniques, possess a highly porous surface, potent regeneration properties, mechanical and environmental effectiveness, and they are less water soluble than other toxic materials [10,17]. PANI-based conjugated CPs easily polymerise with a broad variety of wide gap inorganic semiconducting materials such as halides, sulphides, and oxides of miscellaneous metals which exhibit important photocatalytic, optical, and photoelectric properties [23,24]. Interestingly, under UV irradiation, PANI efficiently acts as an electron donor and supports the transmission holes. The distinguished electron transferring property of PANI composites when irradiated with photons helps to generate and transfer photo generated electrons to the conduction bands of various high band gap semiconducting materials such as titania, zinc oxide, strontium titanate, etc. The high energy of the lowest unoccupied molecular orbital (LUMO) of PANI compared to the metal oxide’s conduction band assists in the electron transfer process [10,25]. Thus, the electron transfer from the CPs to the metal oxide’s CB assists in the deceleration of electron-hole recombination, thereby assisting in the enhancement of the photo response upon photochemical irradiation.

Distinct nanocomposites with attractive properties, obtained by doping inorganic metal oxides, sulphides, nitrides, etc., with CPs, are utilised for photocatalytic reactions, where the semiconducting metal oxides are efficient photosensitisers assisting the photochemical reaction due to the presence of the empty conduction bands (CB) and filled valence bands (VB). PANI, in corroboration with a wide array of metal oxides such as strontium titanate, titania, zinc sulphide, zinc oxide, cobalt oxide, etc., effectively promotes the mobility of the electrons from its excited antibonding orbital ( π* ) to the empty CB of the inorganic oxides and sulphides [26,27,28,29,30]. The electrons and the hole so emitted then reacts with the water and oxygen molecules present in their surroundings to form superoxide and hydroxide radicals which enable the organic molecules to undergo photo degradation. Due to their fundamental and distinct surface properties, two-dimensional semiconductors have been broadly explored in numerous applications [31].

For the fabrication of nanocomposites, inorganic oxides and sulphides are the key constituents to obtain the semiconductor exhibiting superior photoconductivity. Amongst the multifarious inorganic metal oxides and sulphides, tungsten disulphides (WS_2_) are pivotal 2D materials because of their structural similarity to grapheme. Additionally, some of their other distinctive properties include a tolerance to high temperatures, an excellent surface to volume ratio, a good thermal conductance, a wide band gap, a tolerance to chemicals, and superior electron and hole transporting properties [32]. These properties of 2D-WS_2_ assist in outperforming their 0D and 1D counterparts [33].

The versatility of both WS_2_ and PANI inspired us to synthesise 2D WS_2_ incorporated PANI (WS_2_-PANI) nanocomposite materials by an in situ oxidative polymerization technique for its utility in the photochemical degradation of carcinogenic MB dye. The composite materials were synthesised by blending 2D WS_2_ with PANI nanotubes during the in situ aniline polymerization step. The resulting WS_2_-PANI nanocomposite corroborates the properties of both the component’s photoelectron and hole generation, thus aiding in the enhancement of the photocatalytic efficiency of the resulting materials.

## 2. Experimental Section

### 2.1. Materials

Analytical grade ammonium peroxydisulfate (APS) (≥99%) and Aniline (≥99%) were procured from Merck, India while HCl (37%), methanol (99.9%), and acetone (95%) were obtained from Finar, India. All chemicals were used without further purification. Aniline was purified further by distillation under vacuum and reduced pressure followed by storing in dark conditions for further usage. 70 nm WS_2_ powder was obtained from Lower Friction Company (Mississauga, Ontario, Canada). DI water was utilised for all the experiments unless otherwise mentioned.

### 2.2. Preparation of PANI Nanotubes

PANI nanotubes were synthesised by oxidative polymerisation of 0.0215 molar aniline (purified by distillation) using 30 mL 1 M (aq.) HCl and APS were used as oxidants. The catalytic reaction was performed by dropwise addition of APS to aniline under continuous stirring at 0–5 °C. The resulting solution was maintained under stirring conditions for 3 h followed by refrigeration for reaction to proceed to completion. After the reaction’s completion, the resulting solution was filtered followed by washing with 0.5 M HCl to obtain colourless filtrate. Washing of the obtained product was performed using DI water (5 washings), followed by washing with acetone: methanol mixture 1:1 (2 washings) to remove all the unreached monomeric and oligomeric aniline. The final product was kept for drying overnight at 60 °C under 100 mb pressure in a vacuum oven the desired conducing polymer PANI.

### 2.3. Preparation of WS_2_-PANI Nanocomposite

WS_2_-PANI nanocomposites were synthesised by varying the percent weight loadings of WS_2_ nanosheets (1 wt% = WS_2_-PANI-1, 2 wt% = WS_2_-PANI-2, and 5 wt% = WS_2_-PANI-5) with respect to 0.0125 mol of aniline. The well dispersed solution of the aforementioned WS_2_ nanosheets in 5 mL DI water was added dropwise to aniline in HCl solution under constant and high-speed stirring. After the addition was complete, the solution was sonicated for a few minutes until the reaction mixture was uniform. The protocol for WS_2_-PANI processing that was adopted was similar to the procedure mentioned in the previous sections (see Scheme 1).

### 2.4. Characterisation Techniques

To evaluate the surface morphology and structure including the chemical composition of the synthesized product, JEOL JSM-7600F (JEOL Ltd., Tokyo, Japan) field emission scanning electron microscopy (FESEM) functioning at 10 kV was used. High resolution Transmission Electron Microscope (TEM) model JEOL JEM-2100F (JEOL Ltd., Tokyo, Japan) was used to observe the atomic structure, the size, shape, and crystallographic information of the prepared nanocomposites.

As for the thermal stability of the samples, such as degradation temperature, Perkin Elmer TGA6 was employed under nitrogen atmosphere with heating rate of 10 °C/min from room temperature up to 900 °C. In this study, 10 mg dried sample was placed in the alumina crucible, and fluctuations in the mass of the sample was evaluated within the temperature range of 35 °C to 900 °C under N_2_ atmosphere at a flow rate of 30 mL/min. Additionally, the structural characterization of WS_2_-PANI, including crystallinity and phase purity of the WS_2_-PANI, was performed using Empyrean X-ray diffractometer (Malvern Panalytical Ltd., Malvern, UK) exhibiting the X-ray diffraction (XRD) patterns by scanning at the rate of 0.02 s^−1^ and keeping the angle of diffraction 2θ = 10 °C to 90 °C using Cu Kα radiations (λ = 1.5418 Å).

N_2_-adsorption–desorption isotherms of WS2-PANI (1–5) were investigated at 77 K using Micromeritics Tristar II ASAP 2020 (Micromeritics Instrument Corporation GA, USA) WS_2_-PANI. Specific surface areas were calculated using Brunauer–Emmett–Teller (BET) methods. FT-IR spectra of WS_2_-PANI (1–5) nanocomposites were recorded using the Perkin Elmer RX1 FT-IR ATR spectrometer (Perkin Elmer, Billerica, MA, USA) within 400–4000 cm^−1^ range using the KBr pellets.

### 2.5. Measurement of Photocatalytic Activities

The photocatalytic activity of WS_2_, PANI, and WS_2_-PANI (1–5) was used to evaluate the photochemical degradation of aqueous phase methylene blue (MB) dye. The photocatalytic reaction was performed by using 20 mg of photocatalyst dispersed in 100 mL dye solution with an initial concentration of 10 mg L^−1^ in a quartz vessel. Initially, the attainment of adsorption–desorption equilibrium was performed in dark conditions under constant stirring for 60 min. The photocatalytic degradation of MB was performed for all the photocatalysts in the presence of UV-irradiation placed at 3 cm from the light source. The photocatalyst was uniformly dispersed in the reaction mixture by bubbling air under continuous stirring. An amount of 3 mL of dye solution was periodically removed from reaction, centrifuged, and investigated by UV-visible spectroscopy in a quartz cuvette of 1 cm for the kinetic measurement.

## 3. Results

The as prepared WS2-PANI were fully characterised using various physicochemical techniques.

### 3.1. Morphological Analysis of Nanocomposites

**Figure 1:**
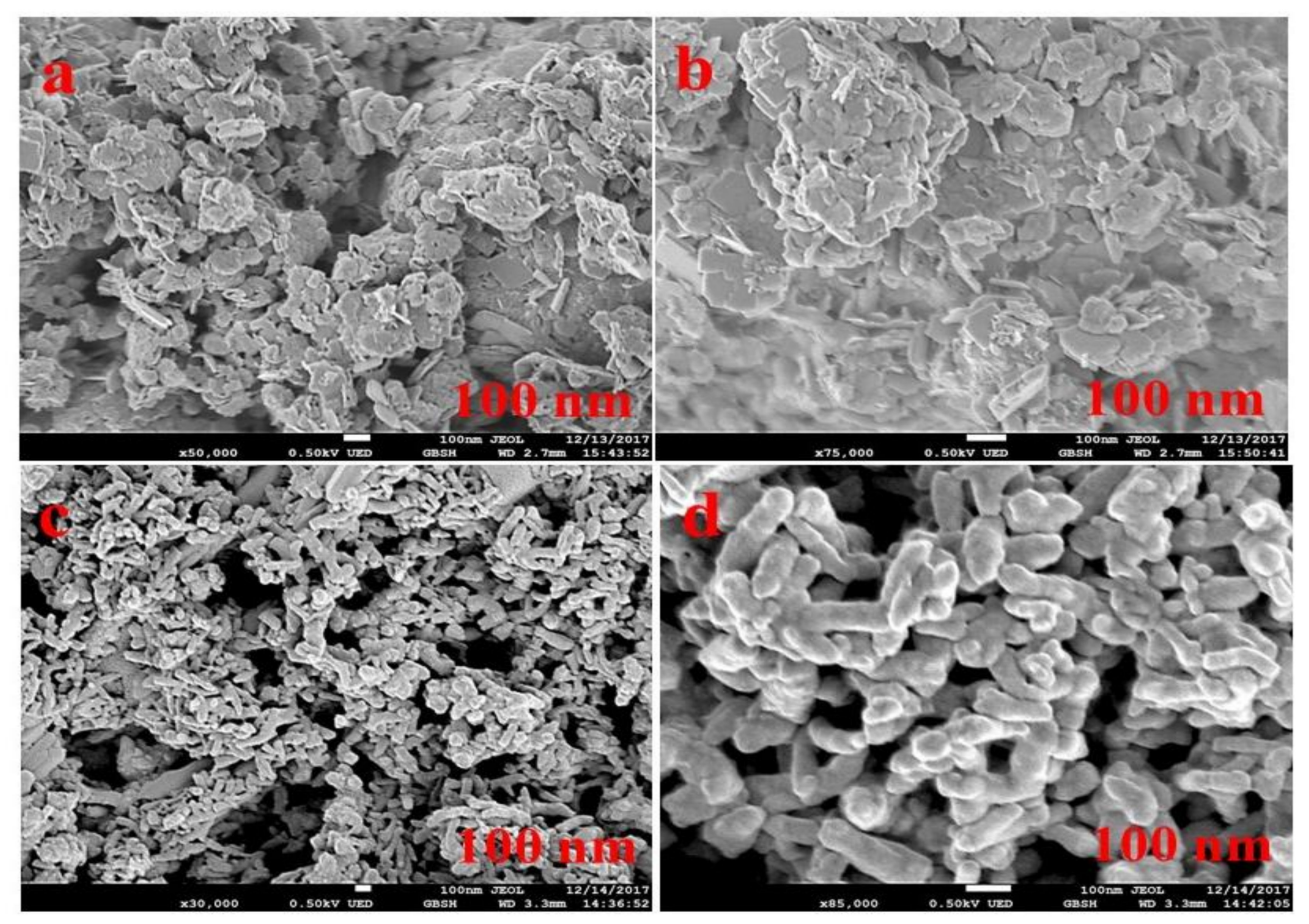
FESEM images of (**a**,**b**) WS_2_ nanoflakes, (**c**) PANI nanotubes, (**d**) WS_2_-PANI granular structure.

The elemental composition and morphological characteristics of the WS_2_, PANI, and WS_2_-PANI (1–5) nanocomposites were extensively investigated using FESEM-EDAX and HRTEM techniques (see Figure 1a,b). The FESM images confirm that the WS_2_ had 2D-nanoflakes with a highly stacked structure. Furthermore, the FESM revealed that PANI (see Figure 1c) had a nanotubular structure, which upon doping with WS_2_ gradually transformed into a granular polymeric network (Figure 1d). Doping PANI polymer with WS_2_ nanosheets did not significantly affect the morphology but did promote the formation of a granular structure (see Figure S1 from the Supplementary Materials). However, an examination of the WS_2_ nanosheets was a daunting task because of their low concentration in the composite materials compared to the PANI polymer.

**Figure 2:**
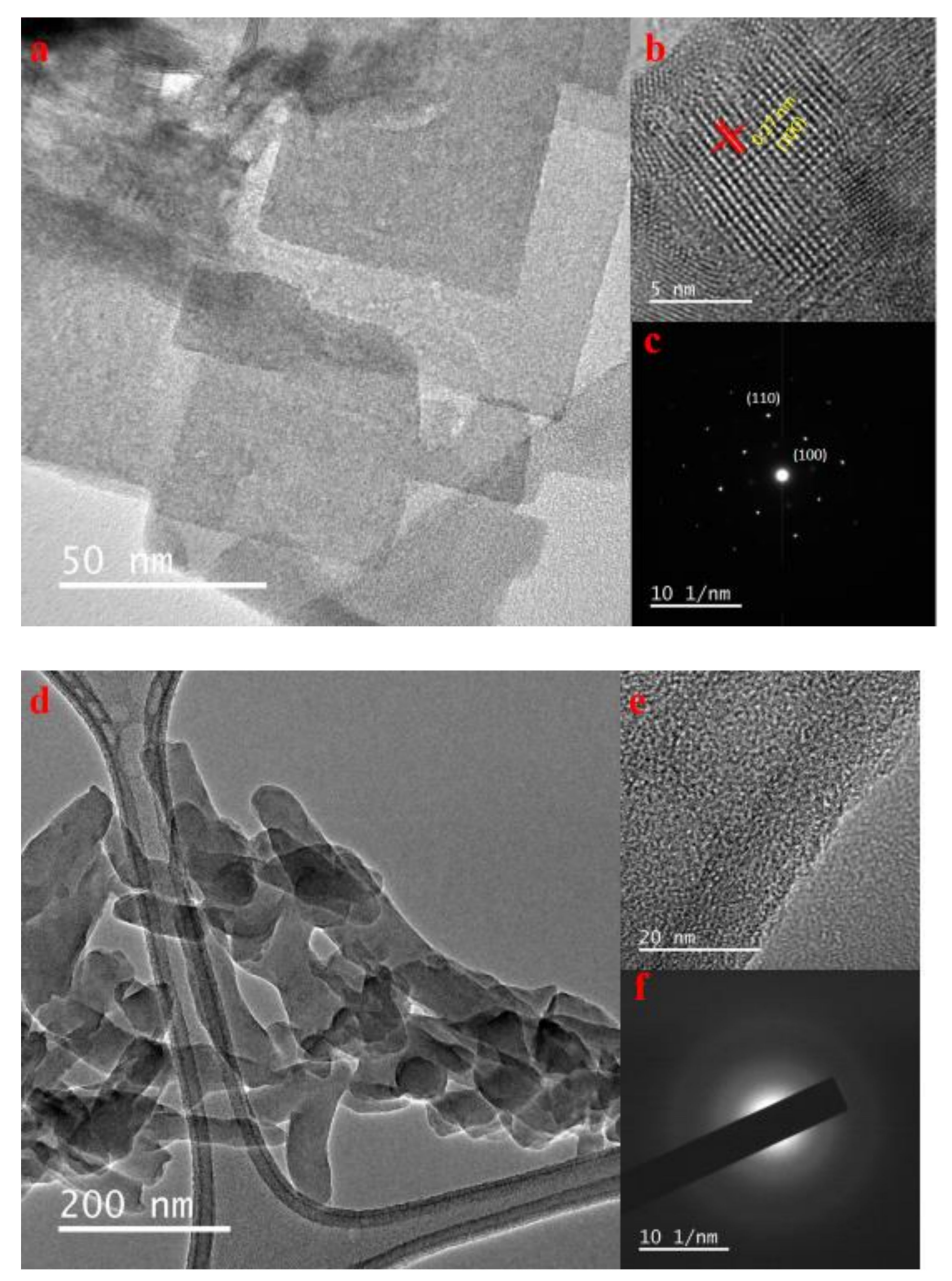
HRTEM images of (**a**) bare WS_2_, (**b**) bare WS_2_, (**c**) SAED pattern for bare WS_2_, (**d**) PANI nanotubes, (**e**) PANI nanotubes, (**f**) SAED pattern for bare PANI.

**Figure 3:**
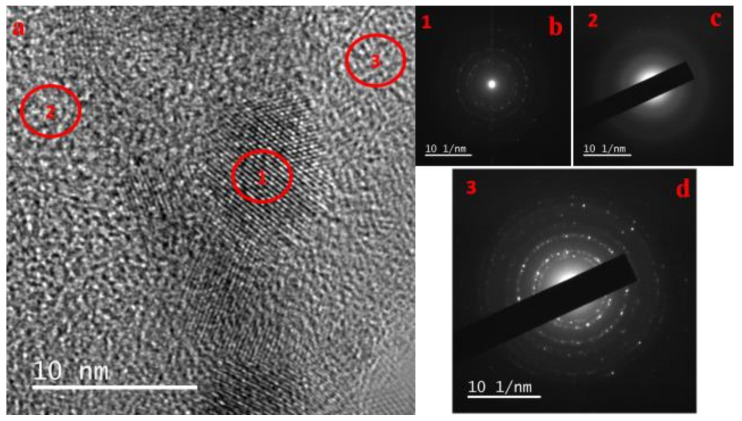
HRTEM images of (**a**) WS_2_-PANI-5 at 10 nm, (**b**–**d**) SAED pattern of WS_2_-PANI-5 of three different spots 1, 2, and 3.

Similar to the FESEM analysis, HRTEM imaging of all the bare and nanocomposite materials (WS_2_, PANI, and WS_2_-PANI) was performed to investigate the atomic arrangement at the nanoscale (see Figure 2 and Figure 3). HRTEM imaging of bare the WS_2_ confirmed the formation of sheet-like layered structure with periodic planes (100) and each layer separated by 0.27 nm (see Figure 2a,b and Figure S2 from the Supplementary Materials). Furthermore, the polycrystalline nature of the WS_2_ materials with high-resolution was examined via a selected area electron diffraction (SAED) pattern (see Figure 2c). The planar orientation depicted the lattice fringes for the (100) and (110) planes which are characteristic of hexagonal WS_2_ [34]. The HRTEM and SAED analysis of the bare PANI polymers corroborates the formation of an amorphous nanotubular structure devoid of a crystalline phase (see Figure 2d–f).

Furthermore, the HRTEM and SAED analyses of WS_2_-PANI-5 nanocomposite exhibit amorphous, crystalline, and mixed phases (amorphous and crystalline) corresponding to the PANI, WS_2_, and composite material (WS_2_ + PANI) as depicted by spots 1, 2, and 3 in the HRTEM image, respectively (see Figure 3).

**Figure 4:**
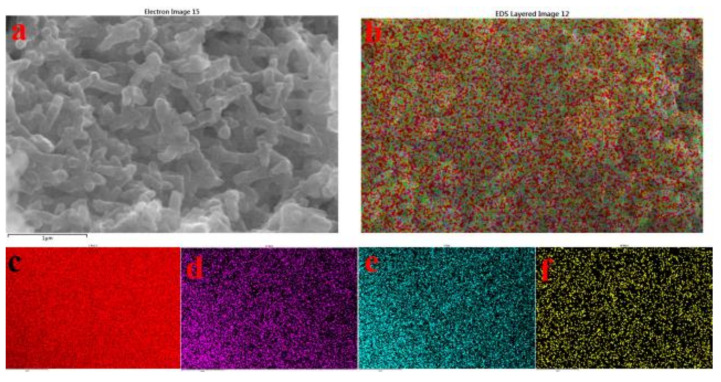
(**a**) FESEM image and (**b**) EDX elemental mapping of WS_2_-PANI-5 nanocomposite on a Si wafer for the following elements: (**c**) C, (**d**) Cl, (**e**) Sulphur, and (**f**) Tungsten.

FESEM or HRTEM analyses alone are not able to depict the presence of WS_2_ nanosheets within the PANI matrix because the WS_2_ was deeply embedded within the polymer matrix and were thus difficult to visualise. Therefore, an FESEM-EDAX elemental analysis was performed on WS_2_-PANI-5 to map the WS_2_ content on the PANI matrix as presented in Figure 4. The EDX analyses clearly confirmed that the tungsten and sulphur content was uniformly dispersed within the polymer matrix along with carbon and nitrogen. This mapping analysis has confirmed the uniform distribution of WS_2_ in the formation of WS_2_ nanosheet nanocomposite.

### 3.2. BET Analysis

**Figure 5:**
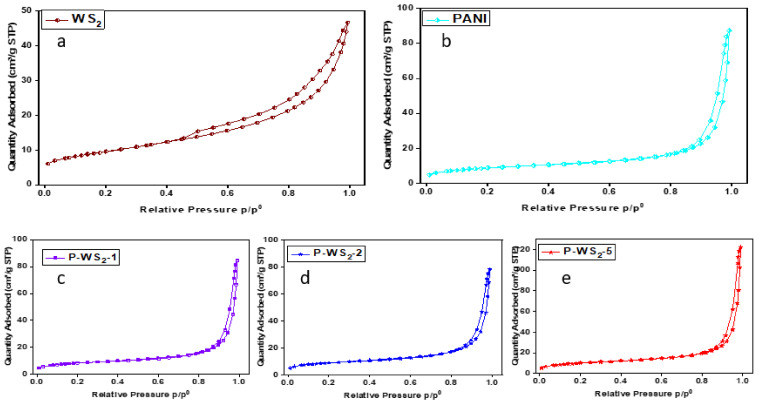
Nitrogen adsorption–desorption isotherms for (**a**) bare WS_2_, (**b**) bare PANI, (**c**) WS_2_-PANI-1, (**d**) WS_2_-PANI-2, and (**e**) WS_2_-PANI-5.

The specific surface area of the bare WS_2_, PANI, and their composite materials was obtained by the Brunauer–Emmett–Teller (BET) technique via nitrogen adsorption–desorption isotherms (see Figure 5). The BET analysis of all the materials exhibit Type-IV isotherms according to the IUPAC classification, corroborating the mesoporous nature of the surface (see Figure 5) [32,35,36]. The BET analysis reveals that the nanocomposites increasing dopant content (1 wt% to 5 wt%) increased the surface area, pore size, and pore volume of the nanocomposite materials compared to bare PANI nanotubes due to variation in the surface morphology (see Table 1). Amongst all the bare and nanocomposite materials, WS_2_-PANI-5 demonstrated the highest surface area and the most porous surface compared to the WS_2_ Nanosheets, PANI nanotubes, PANI-WS_2_-1, and PANI-WS_2_-2. Hence, the BET analysis depicted that the synthesized nanocomposites possess a higher surface area with a porous structure which is a major necessity for an efficient photocatalyst.

### 3.3. XRD Analysis

**Figure 6:**
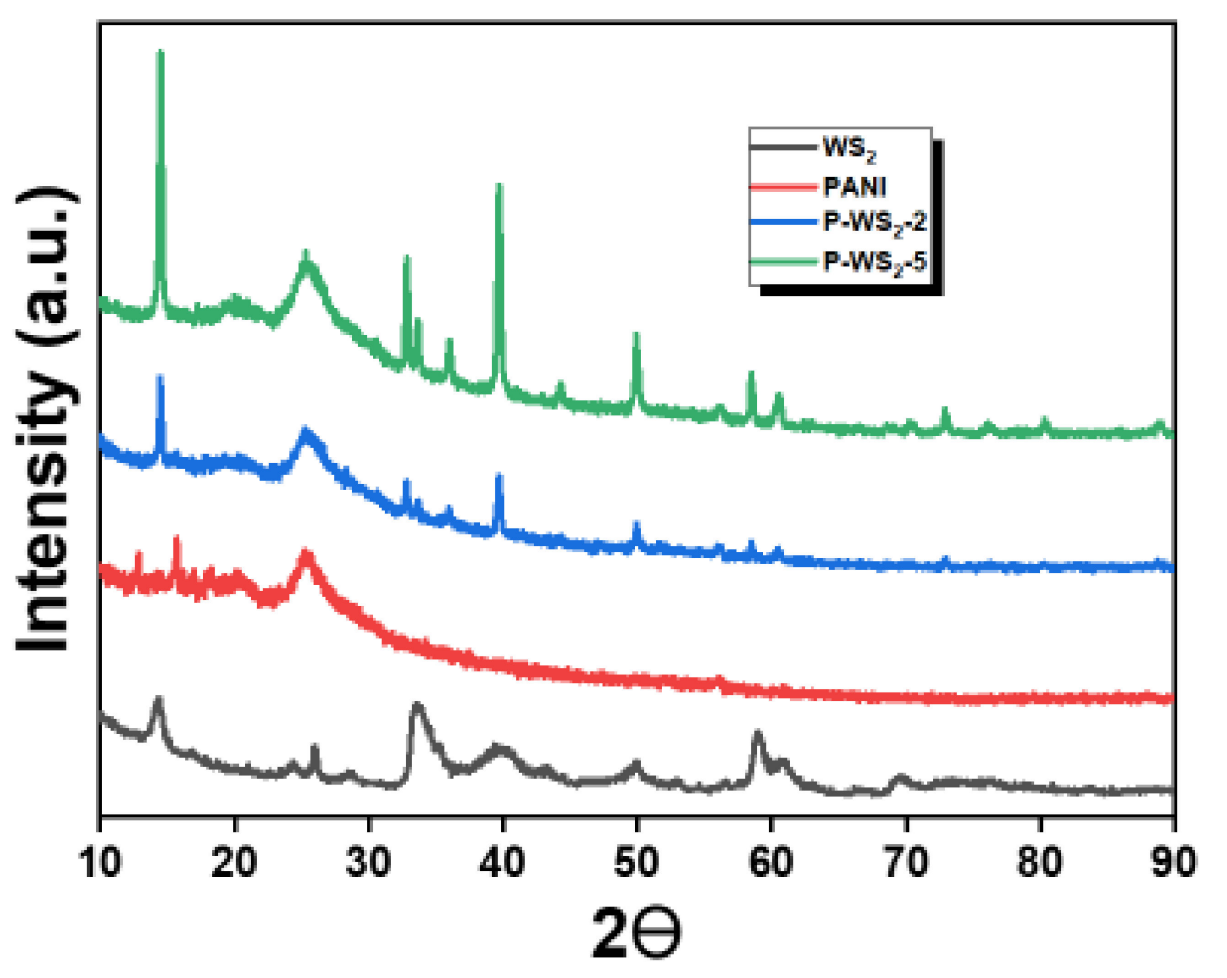
XRD spectra for WS_2_, PANI, WS_2_-PANI-2, and WS_2_-PANI-5.

Figure 6 illustrates the XRD analysis which was performed to analyse the crystal structure of the WS_2_, PANI nanotubes, and WS_2_ nanosheet incorporated nanocomposites. As apparent by the obtained results, the X-ray diffraction pattern for WS_2_ reveals intense and well-defined peaks thus explaining the structural ordering of a higher degree. The characteristic peaks were obtained at 14.35°, 28.89°, 33.56°, 35.94°, 39.52°, 44.01°, 49.80°, and 58.9° which correspond to (002), (004), (101), (102), (103), (006), (105), (106), and (110) standard WS_2_ hexagonal reflection planes as per the JCPDS card No: 841398 [37,38,39]. The intense peak corresponding to the reflection plane (002) represents the stacked layered structured of the 2D WS_2_ nanosheets. As evident from Figure 6, PANI displays the characteristic diffraction peaks at 2θ = 15.66, 20.38, and 25.41, specifying its polycrystalline structure [32]. The characteristic intense peaks at 2θ = 20.38 and 25.41 are probably attributed to the benzenoid and quinoid rings’ periodic repetition, respectively, in the PANI polymeric chains [40].

As obvious from the XRD spectra of the WS_2_ nanosheet incorporated nanocomposites, the characteristic sharp reflection peak of the PANI homopolymer seems to be reduced significantly as it is incorporated with the WS_2_ nanoflakes, and the intensity of this decrement has been more prominent with the increase in the weight percentage of the WS_2_ nanosheets. The characteristic PANI peaks were curtailed due to the presence of the WS_2_ nanosheets which acted as an impurity during the polymerization of PANI and augmented the retardation of the crystalline PANI. Moreover, the characteristic XRD peaks of WS2 in their respective XRD spectra increased with the increasing WS2 content, confirming their presence in the PANI matrix.

### 3.4. FTIR Analysis

**Figure 7:**
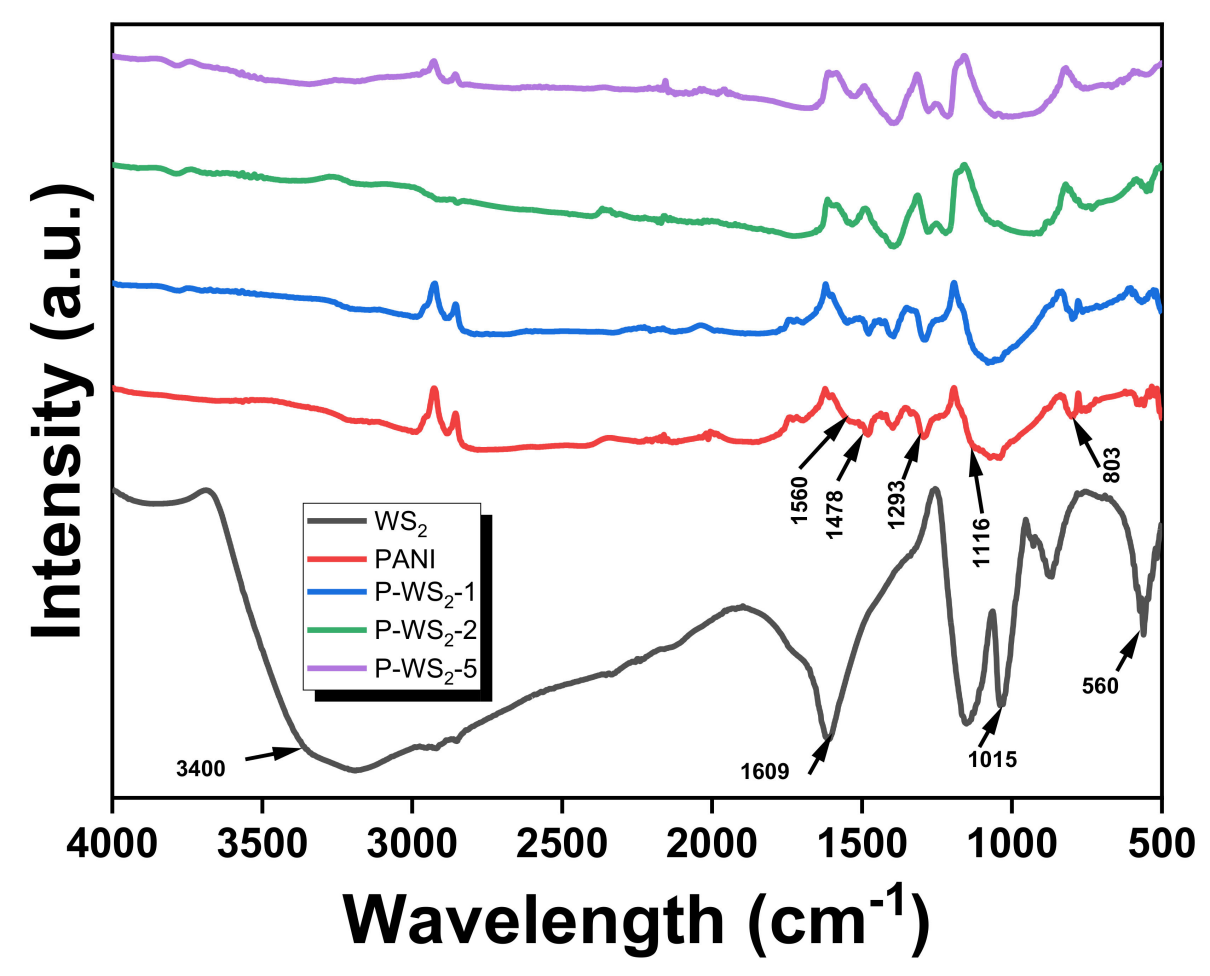
FT-IR spectra of bare WS_2_, bare PANI, and WS_2_-PANI (1–5) nanocomposites.

The FT-IR spectroscopy of all the bare WS_2_, PANI, and their nanocomposite materials’ characteristics are illustrated in Figure 7. As apparent from Figure 7, the band appearing at 560 cm^−1^ was attributed to the W–S bond whereas the band appearing at 1015 cm^−1^ may be assigned to S–S bonds [41]. The obtained sharp peaks at 1609 cm^−1^ may be assigned to the stretching deformation of the hydroxyl groups present in the WS2 framework. Moreover, the vibrational bands appearing at around 3400 cm^−1^ can be ascribed to the atmospheric OH which is due to the adsorbed moisture on the surface of WS_2_ [42].

The spectrum of PANI depicting the fingerprinting peaks of the polymer at around 1560 cm^−1^ and 1478 cm^−1^ are ascribed to the C–C bond’s stretching and deformation of the quinoid and benzenoid rings, respectively [32]. The sharp characteristic peak appearing at 1293 cm^−1^ might be attributed to the C–N and C=N stretching whereas the peaks at 1116 and 803 cm^−1^ are due to the in-plane and out-of-plane bending of C–H bonds in the chains of PANI matrix [32,35]. The FTIR spectra of the nanocomposites incorporated with WS_2_ nanosheets reveal the characteristic peaks of both PANI nanotubes and WS_2_ nanosheets. As evident from the obtained spectra of the nanocomposites, the peak at 1116 cm^−1^ in the PANI homopolymer appeared to be slightly shifted in the nanocomposites. This shifting may be due to the formation of weak Van der Waals bonds between the polymer and nanoparticles. Moreover, the appearance of the characteristic peak of WS_2_ in the nanocomposites (as marked by red arrows) confirmed the doping of the PANI nanotubes with the WS_2_ nanosheet nanocomposite surface.

### 3.5. Photocatalytic Degradation of MB under UV Irradiation

**Figure 8:**
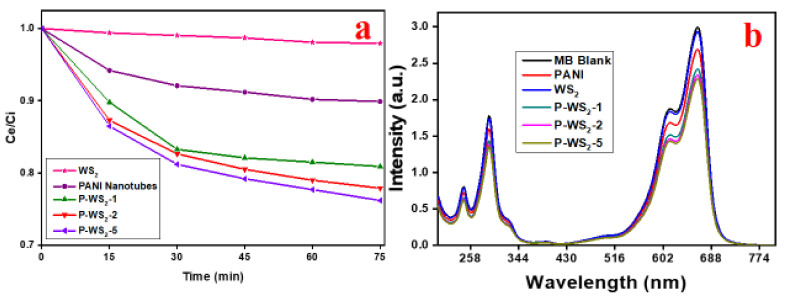
(**a**) Adsorption–desorption equilibrium rate of MB under dark conditions versus time in the presence of various photocatalysts (**b**) UV-vis absorption spectra of MB aqueous solution at 75-min dark adsorption–desorption equilibrium.

The bare WS_2_ nanosheets, PANI homopolymer, and WS_2_-PANI (1–5) nanocomposites were employed for the photocatalytic degradation analysis of methylene blue (MB) in the presence of UV-light illumination at ambient temperature. The dark adsorption–desorption phenomenon was investigated for MB adsorption over the surface of different photocatalysts for 75 min by monitoring the characteristic MB peak in UV-vis analysis. The dark adsorption–desorption equilibrium spectra for all the aforementioned photocatalytic materials confirm that the adsorption of MB on the photocatalysts surface increased with time until an equilibrium was attained after 30 min, as evident from Figure 8. The surface adsorption of MB after the attainment the of dark adsorption–desorption equilibria was found to be 2.05%, 10.1%, 19.1%, 22.1%, and 23.8% for WS_2_ nanosheets, PANI nanotubes, PANI-WS_2_-1, PANI-WS_2_-2 and PANI-WS_2_-5 photocatalysts, respectively. The probable reason for the adsorption of MB on the PANI nanocomposite’s surface was due to the π–π and electrostatic interactions between the polymeric chains of PANI and benzene containing aromatic rings of MB molecules. These interactions aided the adsorption of the dye molecules onto the surface of the nanocomposites thereby augmenting the process of photocatalysis. In addition, the nanocomposites incorporated with WS_2_ nanosheets demonstrated an enhanced adsorption compared to bare the PANI nanotubes or WS_2_ nanosheets. Furthermore, WS_2_-PANI-5 outperformed other composite materials, thus demonstrating its maximum adsorption efficacy.

**Figure 9:**
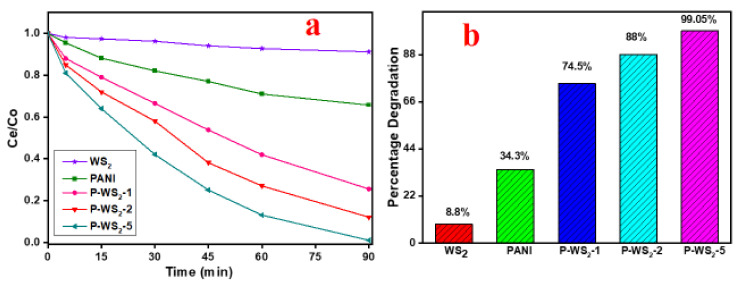
(**a**) Adsorption–desorption equilibrium rate of MB under dark conditions versus time in the presence of various photocatalysts and (**b**) UV-vis absorption spectra of MB aqueous solution at 75-min dark adsorption–desorption equilibrium.

Figure 9 represents the rate of photodegradation and the percentage of the photodegradation of the MB for WS_2_ nanosheets, PANI nanotubes, and WS_2_-PANI (1–5) nanocomposites at different time intervals. The kinetics curves for the photocatalytic degradation of MB using WS_2_-PANI (1–5) nanocomposites demonstrated an improved photocatalytic degradation compared to the bare PANI nanotubes and WS_2_ nanosheets. The photodegradation, as evident form Figure 9b, exhibited the following tendency: WS_2_-PANI-5 > WS_2_-PANI-2 > WS_2_-PANI-1 > PANI nanotubes > WS_2_ nanosheets. The real time photocatalytic data for MB degradation by all the bare and nanocomposite materials are depicted in Figure S3a–f.

The real time UV curves for the photodegradation reveal the significant enhancement of MB photodegradation by WS_2_-PANI (1–5), corroborating the synergistic enhancement of the photocatalytic performance with time compared to bare WS_2_ and PANI. The kinetic curves for the photocatalytic MB degradation by the WS_2_ nanosheets, shown in Figure S3a–f, demonstrated insignificant photocatalytic activity with merely 8.8% of degradation for 90 min of UV light exposure. Whereas PANI homopolymers exhibited better performance as a photocatalyst with 34.3% of photodegradation after 90 min as depicted in Figure S3b. The photodegradation efficiency of the nanocomposites improved substantially with respect to PANI nanotubes as apparent from the Figure S3c–f. To analyse and compare the photocatalytic performances of each photocatalyst after 90 min of UV exposure, the combined UV-vis spectra of WS_2_, PANI nanotubes, and _WS2_-PANI (1–5) incorporated nanocomposites is represented in Figure S3f. As evident from Figure S3f, WS_2_-PANI-5 outperformed other the photocatalytic materials with 99.05% of MB degradation followed by WS_2_-PANI-2 and WS_2_-PANI-1 with 88% and 74.5% of photodegradation, respectively. This increment in the photocatalytic activity is also supported by the surface analysis as discussed in the previous section (BET analysis), whereby the WS_2_-PANI-5 revealed the maximum surface area compared with other compositions. Thus, the photocatalytic analysis has established WS2-PANI-5 as an optimum nanocomposite which demonstrates the highest degradation activity. The photoinduced π-π ^**˟**^ transitions within the polymeric chains of the PANI homopolymer upon irradiation with UV light may be the prime cause of the MB dye’s degradation [32]. The photocatalytic efficiency of the PANI nanotubes was improved considerably by doping them with WS_2_ nanosheets, thus corroborating the improved electronic and optical properties of the WS_2_-PANI (1–5) nanocomposites.

## 4. Mechanism of Photodegradation

**Figure 10:**
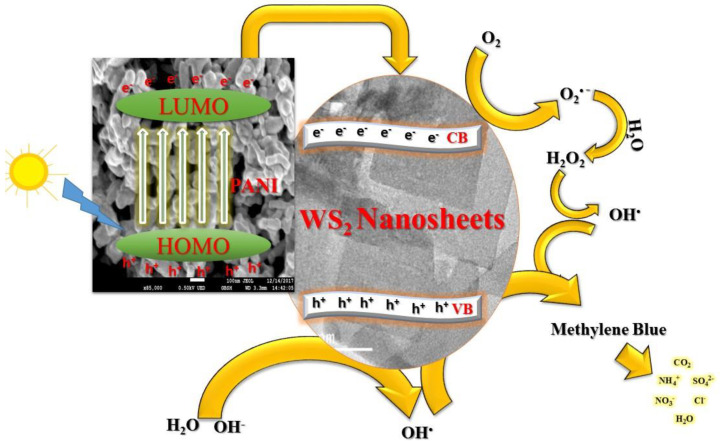
Mechanism for the methylene blue degradation via WS_2_-PNAI-5 nanocomposite materials.

Since MB is a photoactive molecule which has a tendency to absorb visible light within the region of 500–700 nm, it can undergo electronic transitions to form singlet and triplet species leading to its self-decomposition to a certain level [10,35]. The singlets and triplets, formed by the electronic transitions upon the photo illumination, are highly reactive species with extreme energy, which reacts with oxygen molecules forming peroxide, superoxide, and hydroxyl radicals also known as advanced oxidation species (AOS). The AOS are responsible for the degradation of any organic molecule exposed to them and act as scavenger moieties. The AOS formation by the light absorbing dye molecules takes place on a very miniscule scale and is practically insignificant in the self-decomposition of dyes or organic pollutants. This process of the formation of AOS can be substantially enhanced by introducing various photoactive materials which can support the degradation of potential organic molecules. Similar to MB, conductive polymers and various semiconducting materials can also produce AOS which can be tapped to degrade the organic pollutants. Since PANI is a conductive polymer with a positively charged backbone, it can act as an effective electron donor and hole transporter with a higher electron mobility upon exposure to energetic photons. PANI in its conductive state, also known as its emeraldine state, possesses the Highest Occupied Molecular Orbital (HOMO) and Lowest Unoccupied Molecular Orbital (LUMO), which are analogous to the valence and conduction bands, respectively, of conductor and semiconductor materials. The electrons present in the HOMO can be easily excited via characteristic π–π ^**˟**^ transitions upon irradiation with photons and can be transferred to LUMO orbitals [10,12]. These electronic transitions are responsible for the formation of negatively charged electrons and positively charged holes in the HOMO and LUMO orbitals of PANI, respectively, leading to the creation of AOS upon reaction with water and oxygen. These AOS are responsible for the active degradation of organic molecules such as MB dye. However, the formation of electrons and holes is a very short-lived process and the recombination of both takes place very rapidly, limiting the formation of AOS and thereby decreasing the overall efficiency of the bare PANI to act as an efficient photocatalyst [36]. To enhance the photocatalytic efficacy of a conductive PANI homopolymer, it is usually doped with semiconducting material. In this study, PANI has been doped with WS_2_ nanosheets which have a band gap of 1.3–2.2 eV [43] via in situ polymerization process. In WS_2_ doped nanocomposites, the partially filled d-orbitals of the conduction band of WS_2_ undergo electronic interactions with the LUMO of PANI nanotubes upon UV light exposure. The LUMO of PANI nanotubes and the empty d-orbitals of WS_2_ come closer due to the electronic interactions, thereby leading to the transferring of electrons from the LUMO of PANI to the conduction band of WS_2_. This transference of electrons from the LUMO of PANI to the conduction band of WS_2_ prevent the early recombination of holes and electrons, thereby enhancing the formation of AOS. The possible mechanism for the degradation of MB is illustrated in Figure 10. Therefore, WS_2_ and PANI work synergistically and momentously boost the photocatalytic degradation of MB by accelerating the formation of AOS.

## 5. Reproducibility of the Photocatalysts

**Figure 11:**
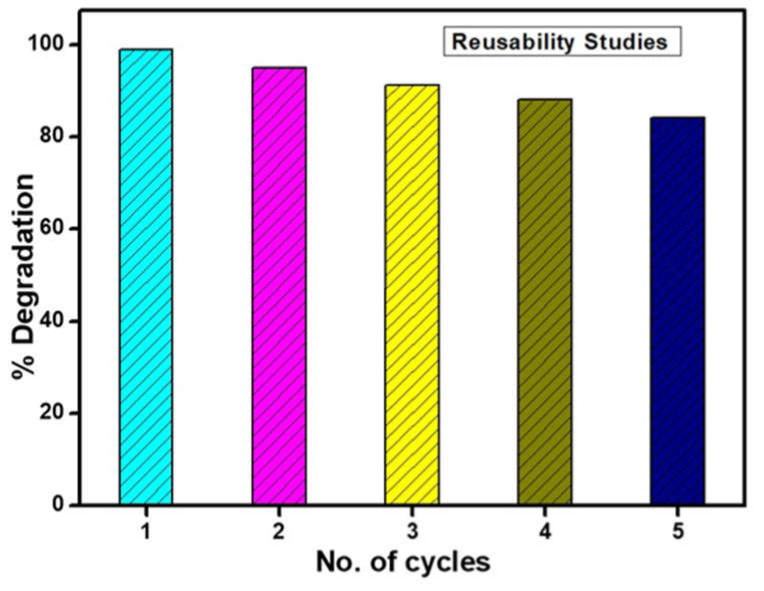
The recyclability study for the WS_2_-PANI-5 based photocatalytic material for the degradation of MB.

To establish sustainable the usability and economic feasibility of a material for potential applications, the reusability of the material is an important parameter which must be investigated for its practical usage. Thus, a reusability examination was done for WS_2_-PANI-5 nanocomposite for MB photodegradation. Figure 11 represents the reusability analysis of WS_2_-PANI-5 for five consecutive cycles. The photocatalyst after every successful photocatalytic degradation cycle was simply recovered via centrifugation and filtration with a subsequent washing with DI water and was finally dried at 80 °C in vacuum oven for overnight. As apparent from Figure 11, there is a marginal decrease in the photocatalytic efficiency of the WS_2_-PANI-5 photocatalyst with every reusability cycle. The reusability results indicated that the obtained photocatalytic efficiency for MB degradation was found to be 99.05%, 95%, 91.35%, 88.10%, and 84.20% for the 2nd, 3rd, 4th, and 5th cycle, respectively. Therefore, the obtained results reveal that even after the fifth cycle, 84% of the dye can be degraded by the photocatalyst indicating the higher structural stability and reusable tendency of the nanocomposite.

### Comparison of Photocatalytic Efficiencies

The results obtained for the photocatalytic performance of WS_2_-PANI-5 were compared with other reported work and summarized in Table 2. In the present investigation, 99.05% photocatalytic degradation of MB was achieved, with a concentration of 20 mg/100 mL of WS_2_-PANI-5 when loaded onto MB with an initial concentration of 10 ppm. The following table shows that WS_2_-PANI-5 exhibits an enhanced photocatalytic activity towards the degradation of MB in an interval time of 90 min. The synthesized nanocomposite has the potential for the efficient treatment of contaminated water, thus addressing the challenges of environmental pollution.

## 6. Conclusions

To summarise, WS_2_-PANI-5 nanocomposite was synthesised via the in situ oxidative polymerisation route. The developed nanocomposite has shown an enhanced degradation of MB dye as an active photocatalyst. The morphological characterisations show that the nanocomposite has a higher surface area with an adequate thermal stability thus contributing efficiently to the effective degradation of water contaminants. Furthermore, the incorporation of WS_2_ nanosheets with PANI nanotubes has shown their synergistic photocatalytic abilities towards MB dye degradation by providing a charge separation phenomenon. The WS_2_-PANI-5 nanocomposite demonstrated a higher degree of MB dye degradation in a short time interval compared to the unaided PANI nanotubes and WS_2_. The UV analysis also confirmed the stability of the synthesised nanocomposite even after a 5th cycle and confirmed that it can still degrade the dye effectively by confirming the reusability of the photocatalyst for a longer duration. The current approach is cost effective, reproducible, and its methodology can be used to further develop the nanocomposites by using the in situ polymerization of metal oxides and metal sulphide materials with conducting polymers, which can in turn be further used to confront the growing threats of environmental pollution.

## Figures and Tables

**Table 1:** BET and BJH Adsorption Cumulative Pore Volume analysis of WS_2_ Nanosheets, PANI Nanotubes, and nanocomposites.

BET Analysis				
S.No.	Material	BET Surface Area (m^2^g^−1^)	Pore Size Å	Pore Volume cm^3^/g
1	WS_2_ Nanosheets	34	85	0.07
2	PANI Nanotubes	30	152	0.07
3	WS_2_-PANI-1	32	173	0.12
4	WS_2_-PANI-2	33	177	0.13
5	WS_2_-PANI-5	36	210	0.19

**Table 2:** Comparative study of the photocatalytic efficiencies of the different photocatalysts.

Photocatalyst	Model Dye	Conc. of Dye (mgL^−1^)	Amount of Catalyst (mg/mL)	% Degradation	Degradation Time (min.)	Source of Light	Ref.
Graphene/ZnS	Methylene Blue	10	0.2	95	180	UV	[26]
Chitosan/PANI/Co_3_O_4_	Methylene Blue	10	0.3	88	180	UV	[12]
PANI/TiO_2_/SiO_2_ membrane	Methyl Orange	1.5	-	87	90	Visible light	[44]
PANI/ZnO	Methylene Blue	3.2	0.4	99	300	Sunlight	[45]
TiO_2_-PANI/Cork	Methyl Orange	15	1	95	210	Natural sunlight	[46]
PANI/SrTiO_3_	Methylene Blue	10	0.3	97	90	Visible light	[10]
Urachin like CuCo_2_O_4_	Methyl Orange	10	0.6	100	240	Sunlight	[47]
Hollow CoFe_2_O_4_ –PANI nanofibers	Methyl Orange	20	0.2	85	120	LED	[48]
rGO–TiO_2_,	Methylene Blue	10	0.125	100	180	Sunlight	[49]
PANI/WS_2_-5	Methylene Blue	10	0.2	99.05	90	UV	This work

## Data Availability

Not applicable.

## Supplementary Materials

The following supporting information can be downloaded at: https://www.mdpi.com/2079-4991/12/12/2090/s1, Figure S1: FESEM micrographs of PANI-WS_2_-5 at higher magnification; Figure S2: HRTEM images of WS_2_ nanosheets depicting molecular fringes; Figure S3: (a–f). UV-vis absorption spectra of MB aqueous solution at different times in the presence of (a) WS_2_ nanosheets (b) PANI Nanotubes (c) PANI-WS_2_-1 (d) PANI-WS_2_-2 (e) PANI-WS_2_-5 and (f) UV-vis absorption spectra of MB photodegradation at 90th minute in presence of different photocatalyst.
