# Supplementary material for: Synthesis and Characterization of 2D-WS2 Incorporated Polyaniline Nanocomposites as Photo Catalyst for Methylene Blue Degradation
Source: Nanomaterials (Basel). 2022 Jun 17;12(12):2090. doi: 10.3390/nano12122090 (PMC9254741; doi:10.3390/nano12122090)
Supplement: Supplementary file 1 [file nanomaterials-12-02090-s001.zip › nanomaterials-1735647-supplementary.pdf]

nanomaterials

Supplementary Materials

Synthesis and Characterization of 2D-WS2 Incorporated Polyaniline Nanocomposites as Photo Catalyst for Methylene Blue Degradation

Syed Shahabuddin [1,*], Shahid Mehmood [2], Irfan Ahmad [3] and Nanthini Sridewi [4,*]

1 Department of Chemistry, School of Technology, Pandit Deendayal Energy University, Raisan, Gandhinagar 382426, Gujarat, India
2 School of Bio-Chemical Engineering and Technology, Sirindhorn International Institute of Technology (SIIT), Thammasat University, PathumThani 12121, Thailand; shahid.mehmoodawan1@gmail.com
3 Department of Clinical Laboratory Sciences, College of Applied Medical Sciences, King Khalid University, Abha 61421, Saudi Arabia; irfancsmmu@gmail.com
4 Department of Maritime Science and Technology, Faculty of Defence Science and Technology, National Defence University of Malaysia, Kuala Lumpur 57000, Malaysia
* Correspondence: syedshahab.hyd@gmail.com or syed.shahabuddin@sot.pdpu.ac.in (S.S); nanthini@upnm.edu.my (N.S.); Tel.: +91-8585932338 (S.S); Tel.: +60-124-675-320 (N.S.)

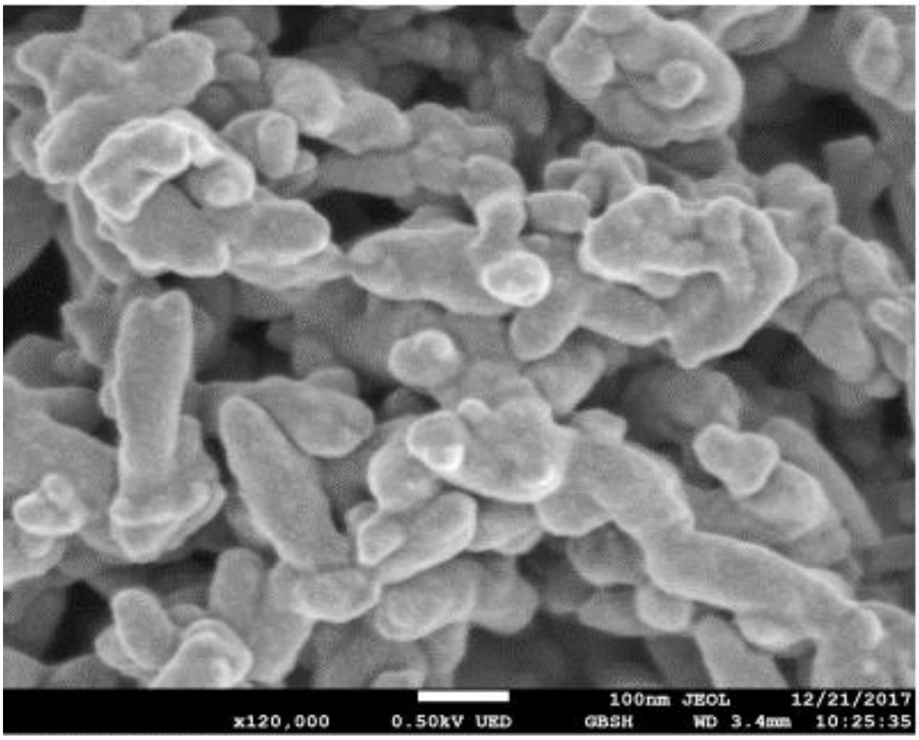

Figure S1. FESEM micrographs of PANI-WS_2-5 at higher magnification.

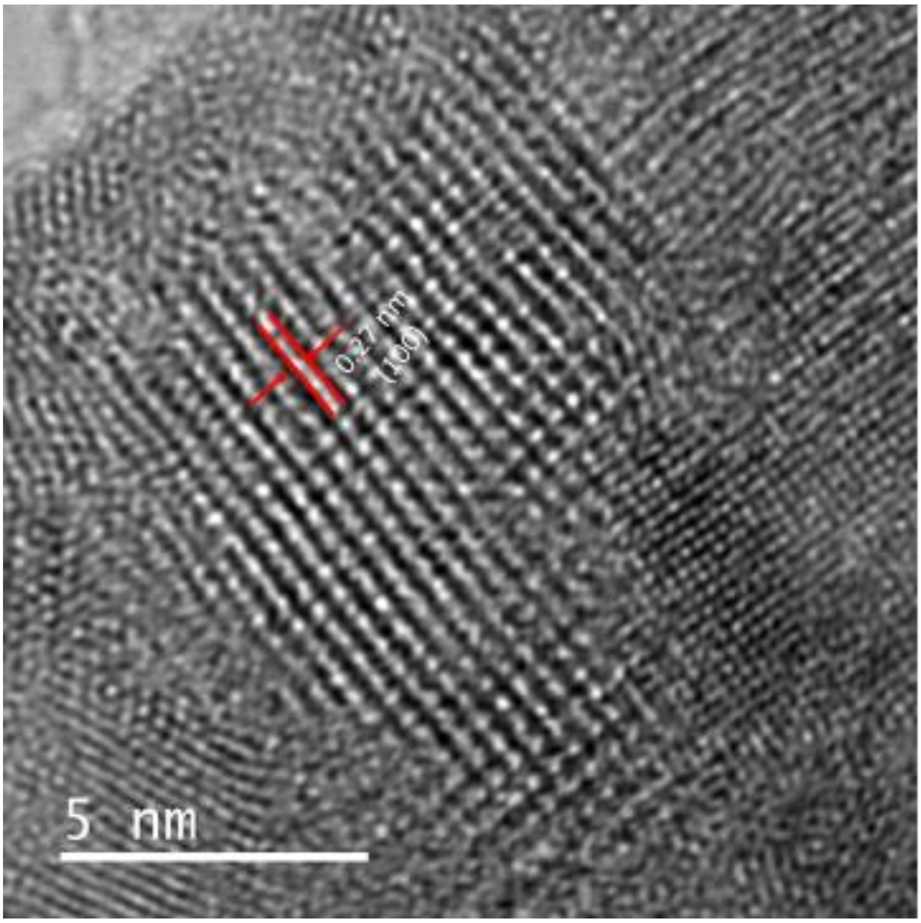

Figure S2. HRTEM images of WS_2 nanosheets depicting molecular fringes.

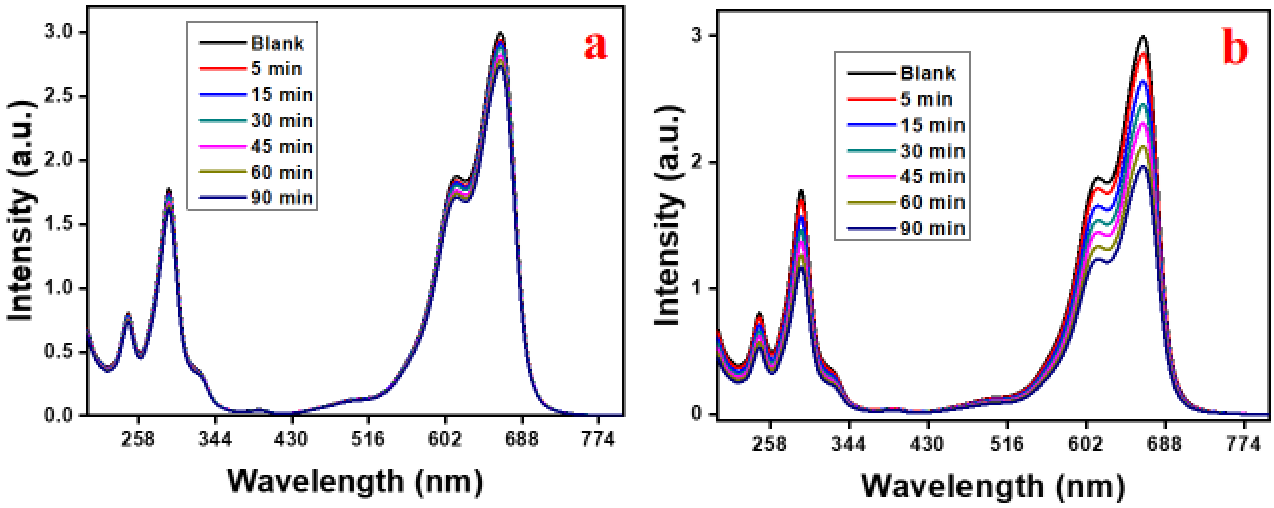

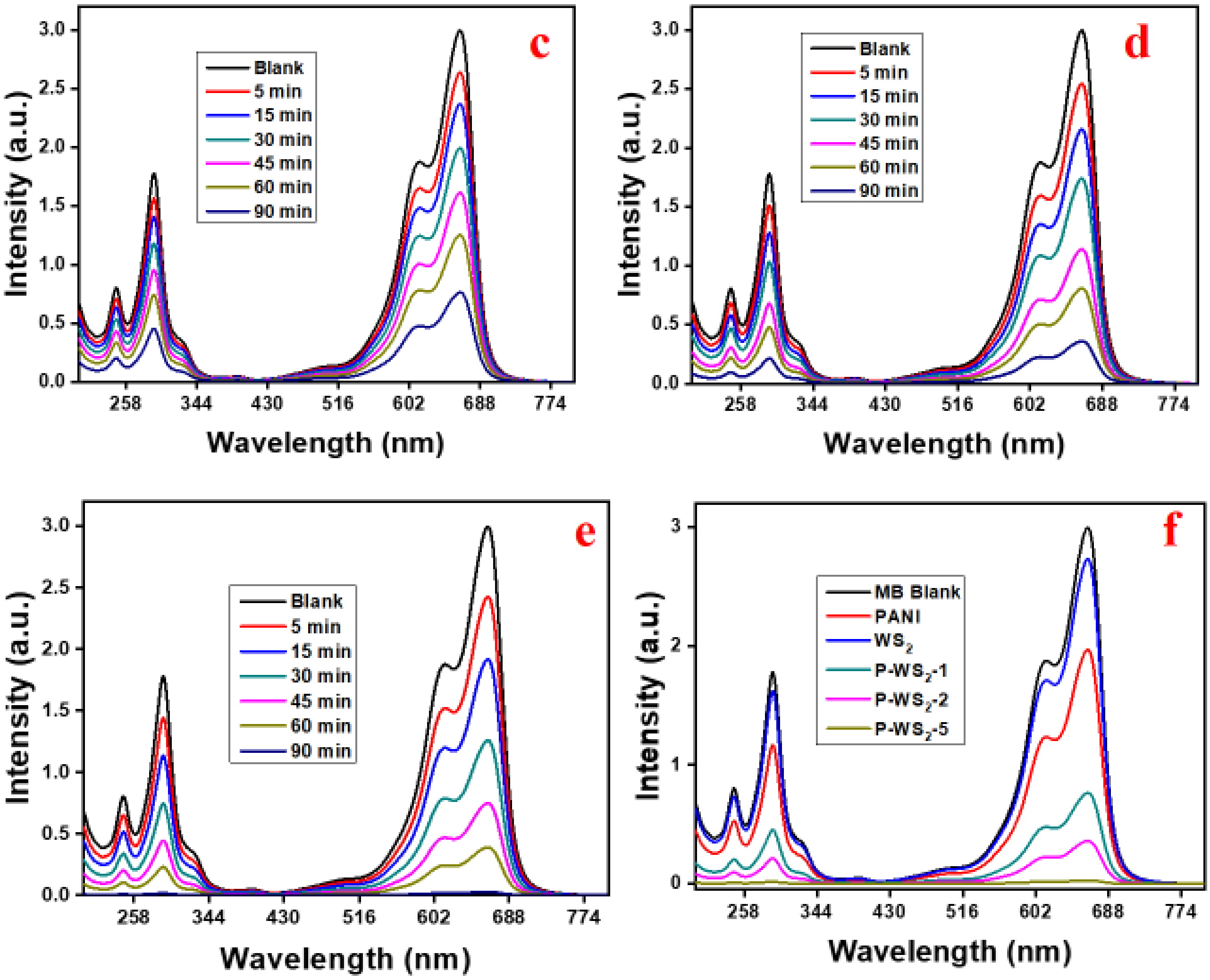

Figure S3. (**a**–**f**). UV-vis absorption spectra of MB aqueous solution at different times in the presence of (**a**) WS_2 nanosheets (**b**) PANI Nanotubes (**c**) PANI-WS_2-1 (**d**) PANI-WS_2-2 (**e**) PANI-WS_2-5 and (**f**) UV-vis absorption spectra of MB photodegradation at 90th minute in presence of different photocatalyst.
